# A Comprehensive Review of Micro-Inertial Measurement Unit Based Intelligent PIG Multi-Sensor Fusion Technologies for Small-Diameter Pipeline Surveying †

**DOI:** 10.3390/mi11090840

**Published:** 2020-09-07

**Authors:** Lianwu Guan, Xiaodan Cong, Qing Zhang, Fanming Liu, Yanbin Gao, Wendou An, Aboelmagd Noureldin

**Affiliations:** 1 College of Intelligent Systems Science and Engineering, Harbin Engineering University, Harbin 150001, China; guanlianwu@hrbeu.edu.cn (L.G.); hrblfm407@hrbeu.edu.cn (F.L.); gaoyanbin@hrbeu.edu.cn (Y.G.); 2 Institute of Intelligent Manufacturing, Heilongjiang Academy of Sciences, Harbin 150008, China; congxiaodan@haai.com.cn; 3 Department of Intelligent Manufacturing and Industrial Safety, Chongqing Vocational Institute of Safety & Technology, Chongqing 404020, China; awd13038301108@aliyun.com; 4 Department of Electrical and Computer Engineering, Royal Military College of Canada, Kingston, ON K7K 7P7, Canada; Aboelmagd.Noureldin@rmc.ca

**Keywords:** pipeline inspection gauge, small-diameter pipeline, pipeline integrity management, intelligent Pipeline Inspection Gauge surveying technology, multi-sensor fused system

## Abstract

It is of great importance for pipeline systems to be is efficient, cost-effective and safe during the transportation of the liquids and gases. However, underground pipelines often experience leaks due to corrosion, human destruction or theft, long-term Earth movement, natural disasters and so on. Leakage or explosion of the operating pipeline usually cause great economical loss, environmental pollution or even a threat to citizens, especially when these accidents occur in human-concentrated urban areas. Therefore, the surveying of the routed pipeline is of vital importance for the Pipeline Integrated Management (PIM). In this paper, a comprehensive review of the Micro-Inertial Measurement Unit (MIMU)-based intelligent Pipeline Inspection Gauge (PIG) multi-sensor fusion technologies for the transport of liquids and gases purposed for small-diameter pipeline (D < 30 cm) surveying is demonstrated. Firstly, four types of typical small-diameter intelligent PIGs and their corresponding pipeline-defects inspection technologies and defects-positioning technologies are investigated according to the various pipeline defects inspection and localization principles. Secondly, the multi-sensor fused pipeline surveying technologies are classified into two main categories, the non-inertial-based and the MIMU-based intelligent PIG surveying technology. Moreover, five schematic diagrams of the MIMU fused intelligent PIG fusion technology is also surveyed and analyzed with details. Thirdly, the potential research directions and challenges of the popular intelligent PIG surveying techniques by multi-sensor fusion system are further presented with details. Finally, the review is comprehensively concluded and demonstrated.

## 1. Introduction

At present, pipeline transportation systems are one of the safest, cost-effective and efficient transportation tools in comparison with conventional land-based, marine, air and railway transportation [1,2,3]. There are over 4 million kilometers (over 600 thousand kilometers in China) of pipelines buried under the Earth and marine seabed, and the diameter of at least 1/3 of the total pipelines is less than 12 inches (small-diameter pipeline, D<30cm) [4,5,6]. Generally, Pipeline Integrated Management (PIM) should be strictly conducted to ensure that the operating pipeline has a safety rate of 99.9%, computed accordingly to [7,8]. Corrosion, human activities, long-term Earth movement and natural disasters are the main reasons for an operation pipeline to be cracked, crooked, leakage or in the event of an explosion [9,10]. Leakages of transported energy result in environmental pollution, economic losses, energies and a waste of resources. Worse is the leakage or the explosion of the operating pipeline in a human-concentrated area, which is a potential threat to human lives, which would lead to a significant negative influence on society [11,12]. Therefore, to implement the PIM rigidly on the operating pipelines is of great importance to maintain the safety of the pipeline transportation system and remain beneficial to human beings.

Generally, there are usually three types of pipelines including the existing pipeline, the newly trenched pipeline and the trenchless pipeline. They are classified based on the pipeline surveying technologies underground, both in land and marine conditions [13]. The advantages of the buried pipelines, which can be regularly surveyed by the intelligent Pipeline Inspection Gauges (PIGs), are shown in Table 1 in comparison to conventional pipeline surveying technologies [14,15]. Firstly, the traditional optical surveying methods, such as the land optical station surveying system, the underwater Remote Operated Vehicle (ROV) and the Autonomous Underwater Vehicle (AUV) systems, are only suitable for the newly trenched pipelines before they are completely buried under the Earth or under the seabed [15]. Secondly, both the ground penetrating radar system and the walkover beacon system are useful for the shallow-depth buried existing and trenchless pipelines. However, these pipelines are not viable for the newly trenched pipelines because of their high cost when compared with the traditional optical surveying method. Finally, the multi-sensor fused surveying method of the intelligent Pipeline Inspection Gauge (PIG) is valid and effective for the inspection and localization of all three types of pipelines as it is not limited by the external environment of the buried pipeline [16]. Moreover, the inspection efficiency of the intelligent PIG is much higher than the conventional surveying technologies [17]. Except for the pipeline surveying technologies in Table 1, there are also some sensors, such as the fiber sensor, acceleration sensor installed on the inner or outer surface of the pipeline to surveil the real-time conditions of the operating pipeline [18,19]. However, this method can only detect the leakage after it occurs and cannot predict or detect in advance, and it is costly to bury these sensors. All in all, the multi-sensor fused intelligent PIG surveying method is the most properly and widely utilized pipeline surveying technology and therefore it presents the greatest potential to be fully developed and applied [20,21].

There are a dozen types of PIGs used for different pipeline-related tasks [22,23,24,25]. For example, trenching robots are usually utilized for implementing the pipeline routing and burying with high efficiency and safety [22]. The welding robots are adopted for connecting the different straight or bending pipeline segments and can even repair pipeline defects with its mechanical arms [23]. The cleaning and caliper measurement PIGs are applied for cleaning the impurities in the bottom of the inner surface of the pipeline before accurate surveying, and measuring the inner diameter of the inspected pipeline [24]. Finally, the intelligent PIG is used to inspect the pipeline wall defects (corrosion, dents, cracks, pits and so on) and implement the pipeline surveying task. The intelligent PIG is one of the most comprehensive and effective tools that carries the PIG inspection system and PIG surveying system to implement the PIM of the pipelines when it travels inside the inspected pipeline [26,27,28]. Nevertheless, due to the limitations of its dimensions, power consumption and surveying precision of the small-dimension sensors, small-diameter pipeline surveying and inspection technologies have been a research focus in recent years [28].

The inspection system of the intelligent PIG is made up of various inspection sensors according to the inspection principles of different inspection tasks for the pipeline [29,30,31,32]. Specifically, geometric detection, Magnetic Flux Leakage (MFL) detection, ultrasonic detection, eddy current detection, visual inspection and so on are combined on intelligent PIGs to detect the various defects from the inner surface, outer surface and wall of the pipelines [33]. Furthermore, the intelligent PIG inspection system would integrate and analyze the signal from these sensors to provide evidence of pipeline defects and their corresponding time epoch. The surveying system of the intelligent PIG is also comprised of multi-sensors such as odometers, Inertial Measurement Unit (IMU), visual sensors, Global Navigation Satellite System (GNSS), pipeline characteristics and so on to calculate the coordinate information of the calculated pipeline central-line and the corresponding time epoch by multi-sensor information fusion technology [34]. Finally, the results from the inspection system and the surveying system of the intelligent PIG are synchronized by GNSS time epochs to provide the relationship between the pipeline defects and their corresponding coordinate distributions. Therefore, the surveying results of the intelligent PIG are mainly adopted for the PIM and the pipeline defects repair guidance, which has been successfully applied in the large-diameter (D>30cm) and long-distance (usually several hundred to thousand kilometers) pipelines [35].

In this paper, to assist researchers and engineers to fully understand the development and applications of the intelligent PIGs, a comprehensive review of the Micro IMU (MIMU)-based intelligent PIG multi-sensor fusion technologies for small-diameter pipeline surveying is introduced. Specifically, the four types of intelligent PIGs and their corresponding pipeline defect detection methods that are typically used for the small-diameter pipeline surveying is introduced in Section 2. After that, the existing and typical PIG surveying methods and their classification based on the non-inertial and the IMU categories are presented in Section 3. In addition, some of the typical IMUs used for intelligent PIG surveying are also described in Section 3. Thirdly, four kinds of typical and potential methods to improve surveying precision for the MIMU-based multi-sensor fusion small-diameter intelligent PIG surveying technologies are reviewed in Section 4. Finally, the challenges and the potential research directions of the MIMU-based intelligent PIG multi-sensor fusion technologies for small-diameter pipeline surveying are also analyzed and presented in Section 5. Section 6 summarizes the overall idea of the paper.

## 2. Small-Diameter Intelligent Pipeline Inspection Gauges (PIGs) and Its Pipeline Defects Inspection Technologies

### 2.1. Small-Diameter Intelligent PIGs

**Figure 1:**
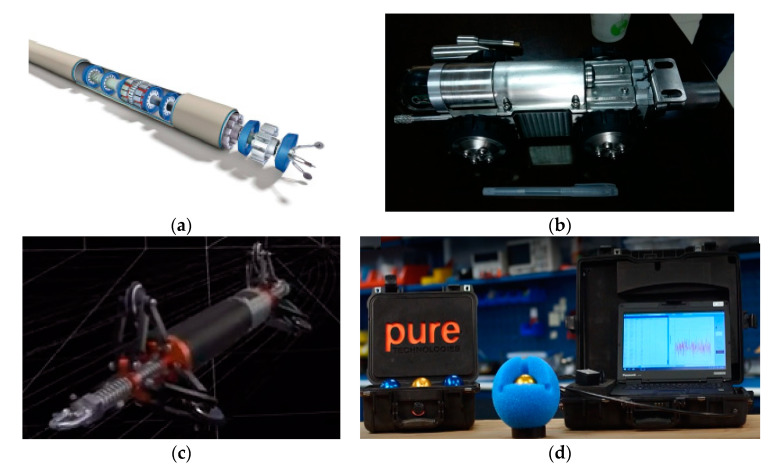
The intelligent Pipeline Inspection Gauges (PIGs) for small-diameter pipeline surveying. (**a**) Regular smart PIG. (**b**) Remote PIG. (**c**) Gyroscopic PIG. (**d**) SmartBall PIG.

Usually, there are four types of intelligent PIGs that are widely used for the PIM of the small-diameter pipeline, which are categorized by the regular smart PIG, the remoted PIG, the gyroscopic PIG and the SmartBall PIG according to their dynamic motion mechanisms for surveying various pipelines [36,37,38,39,40]. These are shown in Figure 1 from (a) to (d), respectively.

In Figure 1a, the regular smart PIG is snake-shaped, which carries the pipeline defect detection sensors and the pipeline surveying sensors by several cylindrical sections, to implement the surveying of the inspected pipelines after it is driven by the pressure differential between the two adjacent ends of the cylindrical sections [26,27,41]. Specifically, the operating interval time and travel distance between each launching and receiving are determined by the installed batteries and the smoothing condition of the inner surface of the inspected pipeline. Moreover, the smallest inner diameter of the pipeline can be as low as 10 cm by using this regular smart PIG. However, the surveying tasks conducted by the regular smart PIG requires the cleaning and caliper PIG to clean impurities and measure the inner diameter of the inspected pipeline carefully and accurately, which would avoid the risk of the smart PIG being blocked into the operating pipeline. More importantly, communication between the regular smart PIG and the aboveground monitor is implemented by the PIG transmitter installed in the PIG end, and the PIG tracking and locating receiver mounted along the outer surface of the pipeline or valves. The rough position of the regular smart PIG is detected once it passes near one of the preset PIG tracking and locating receivers [42].

In Figure 1b, the remote PIG is a cylindrical-shaped robot and moves with four wheels to inspect the impurities in the inner surface of the inspected pipeline by visual sensors [43] This PIG is connected by the fiber communication and power transmission cables to the above ground control and monitor system, and the power supply system as well. Generally, the inner diameter of the inspected pipeline by using the remote PIG can be as small as 10 cm. However, the travel distance of the remoted PIG in the pipeline is limited by the length of the connected fiber cable. The maximum length of the fiber cable would be as much as 200 m for the remoted PIG in small-diameter pipeline surveying application. Meanwhile, the travel distance of the remoted PIG is recorded by the length of the fiber communication and power transmission cable inside of the inspected pipeline [43].

In Figure 1c, the gyroscopic PIG is equipped with high-precision inertial surveying system and symmetric installed odometers on the front and the rear part of the gyroscopic PIG [15]. During the surveying process, it pulls inside the surveyed pipeline by the rope fixed on the front and the rear part of the gyroscopic PIG. The inertial surveying system used to measure the linear acceleration and the angular rate of the PIG in 3D space when the gyroscopic PIG travels inside the pipeline. Meanwhile, the symmetric installed odometers to measure the travel velocity of the PIG in the surveyed pipeline. Finally, all of this information is integrated to calculate the precise trajectory coordinate of the PIG. Furthermore, the travel distance of the gyroscopic PIG is determined by the rope length and the adjacent valves of the surveyed pipeline, which is usually less than 100 m [41]. The trajectory of the inspected pipeline surveying precision is usually less than 1 m because both the online and offline estimation technologies are used to improve the surveying precision of the gyroscopic PIG. Worth noting is that the gyroscopic PIG is not communicated with the aboveground monitor and it is fully controlled by the rope when travelling inside the pipelines [44].

In Figure 1d, the SmartBall PIG is a ball-shaped PIG that travels inside the water pipelines along with the flow of the water [45,46]. It detects the leakages of the water from the pipeline by analyzing the acoustic sensor to transmit and receive the acoustic signal. Moreover, the SmartBall PIG integrates the signals from the accelerometers and the SmartBall PIG above ground markers to calculate the location of the leakages of the pipeline. The detection for each launching and receiving of the SmartBall PIG is usually with the minimum inner diameter of 20 cm, the maximum working period is 12 h, and the maximum travel distance is 48 km. In addition, the SmartBall PIG communicates with the aboveground monitor by using the PIG transmitter and the PIG tracking and locating receiver, which is the same as the regular smart PIG [42]. Finally, the surveying error of the pipeline leakage location by the SmartBall PIG is less than 2 m within two adjacent aboveground markers of the SmartBall PIG.

### 2.2. Pipeline Leakage Inspection Technology

**Figure 2:**
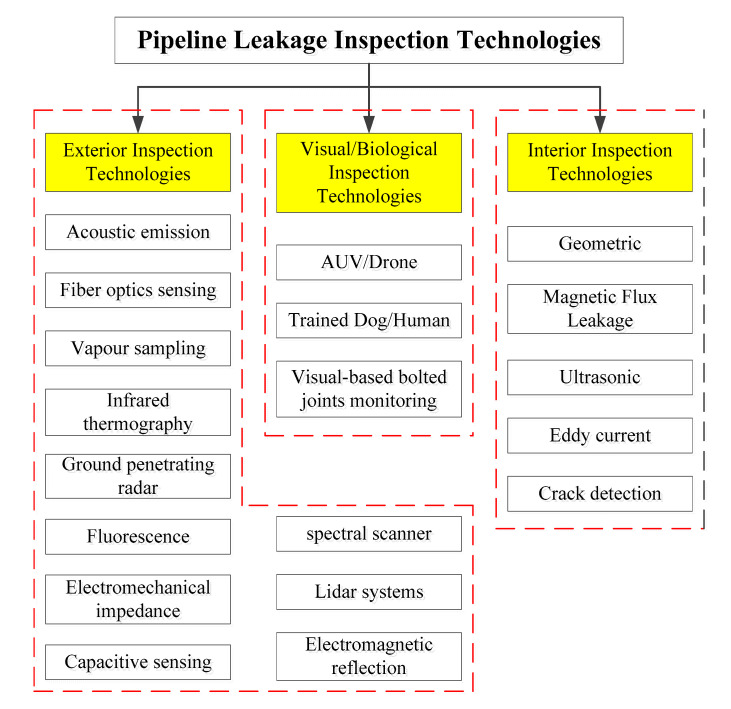
Schematic diagram of different pipeline leakage inspection technologies.

The flow chart of different leakage inspection technologies is shown in Figure 2. There are many kinds of pipeline leakage inspection technologies used for discovering existing and even the potential pipeline defects [47,48,49,50,51]. The pipeline leakage inspection technologies are usually categorized by the exterior inspection technologies, the visual/biological inspection technologies and the interior inspection technologies [45]. Specifically, the exterior pipeline leak detection methods, such as acoustic emission, fiber optics sensing, vapour sampling, infrared thermography, ground penetrating radar, fluorescence, electromechanical impedance, capacitive sensing, spectral scanner, Lidar systems, electromagnetic reflection and so on [46,47]. However, geometric detection, MFL detection, ultrasonic detection, eddy current detection, crack detection and so on are usually utilized to detect various defects from the interior of the inspected pipeline [48,50].

To be more intuitive, Table 2 summarized the pipeline interior defects inspection technologies with their corresponding operation principles, strengths and weaknesses. Moreover, the visual/biological inspection technologies usually implemented by the AUV/Drone, trained dog/human, and even the visual-based bolted joints monitoring, etc. Therefore, the pipeline leakage or defects inspection technologies are comprehensively selected by the actual requirements/tasks and the real conditions of the inspected pipeline.

## 3. PIG Surveying Technologies

The pipeline surveying technology by intelligent PIG is originally from the design and manufacture of the intelligent PIG, and it is usually classified by two main categories according to the key sensors installed in the intelligent PIG during the overall surveying process. This section reviews the non-inertial and the inertial-based intelligent PIG surveying technologies for the PIM during the online inspection.

### 3.1. Non-inertial Based PIG Surveying Technology

**Figure 3:**
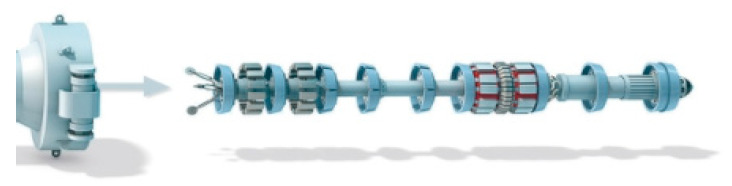
PIG with odometers and transmitter.

The regular smart PIG is a usually designed as a rectangular-shaped robot, which would fit tightly in the inner surface of cylindrical-shaped pipelines. At the beginning of the designing phase, only three symmetrical installed odometers are used as the travel distance measurement equipment of the regular smart PIG in the inspected pipeline, which is shown in Figure 3 [51,52]. However, the distance measurement odometers installed on the regular smart PIG could slip when the regular smart PIG travels over areas covered by wax or mud, and the regular smart PIG travels over the Pipeline Bending Angle (PBA) [53,54]. Therefore, the measurement precision of the travel distance of the regular smart PIG depends on the odometers, which would decrease and accumulate with the travel distance in the pipeline, especially when slippage has occurred.

**Figure 4:**
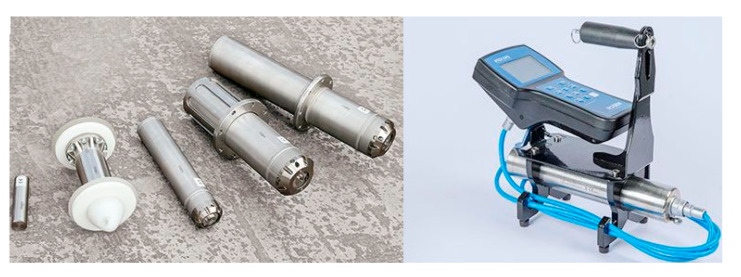
PIG transmitter and PIG tracking & locating receiver.

In addition, the PIG transmitter and the PIG tracking and locating receiver, which are shown in Figure 4, are also integrated to track and locate the PIG between the two adjacent PIG tracking and locating receiver mounted along the pipeline [55,56]. In general, the PIG transmitter is mounted on the rear part of the intelligent PIG for accurate tracking and location within the pipeline. The PIG tracking and locating receivers are installed on the surface of the valves of the pipeline to track and locate the real-time position of the operating PIG. However, PIG tracking and locating receiver would be limited by the aboveground environment of the inspected pipeline. The precise coordinates of the PIG tracking and locating receiver provided by the Differential GNSS (GNSS) would be easily influenced by the surrounding tunnels, forests, high buildings, roads, rivers and so on [57,58].

Nevertheless, the 3D trajectory of the inspected pipeline is not accessible by using the non-inertial-based PIG surveying technology. The 3D trajectory of the inspected pipeline is of vital importance both for the PIM and for the digital and intelligent pipeline management especially when these pipelines are routed under the urban areas and geologically unstable areas [59,60].

### 3.2. Inertial Based PIG Surveying Technology

To improve the surveying precision of the intelligent PIG and avoid the problems of the non-inertial-based PIG surveying technology, the inertial sensors are the best choice to remedy these problems [61]. The inertial base intelligent PIG surveying technology was originally developed by Hanna P.L. at the University of Calgary in Canada in the 1990s [62]. During that period, the tactical-grade Fiber Optic Gyroscope (FOG) comprised of a Strapdown Inertial Navigation System (SINS) is the best choice for the location of the PIG [63,64,65]. Because the traditional large-volume and high-precision mechanical gyroscope comprised Platform Inertial Navigation System (PINS) is not only too large in volume, but has limitations in its measurement range. While the small-size and low-precision micro-electronic mechanical system (MEMS) gyroscope-constructed SINS have benefits in terms of volume, precision, energy-consumption and costs, but are lacking in terms of precision.

**Figure 5:**
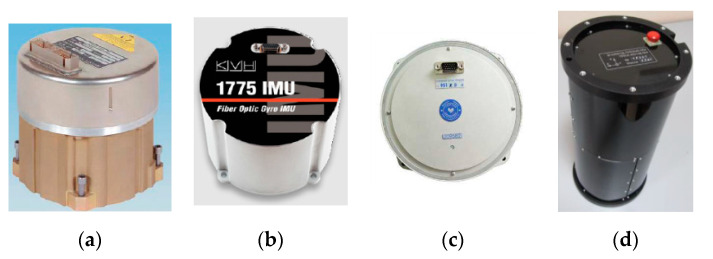
Fiber Optic Gyroscope (FOG) Inertial Measurement Units (IMUs) for intelligent PIG surveying. (**a**) LN-200 IMU. (**b**) 1775 IMU. (**c**) VG-951 FOG. (**d**) IMU-501D.

Currently, the typical tactical-grade FOG-based Inertial Measurement Units (IMUs) used for pipeline surveying in intelligent PIG are the North Grumman LN-200 FOG IMU (Figure 5a), the KVH 1775 FOG IMU (Figure 5b), the Fizoptika VG-951 FOG (Figure 5c) and the Optlink FOG IMU-501D (Figure 5d) [63,64,65]. Therefore, the viable measurement diameter range of these tactical-grade IMUs comprised SINS in the intelligent PIG surveying is usually greater than 30 cm, which cannot be utilized for the intelligent PIG surveying when the inner diameter of the pipeline is less than 30 cm.

**Figure 6:**
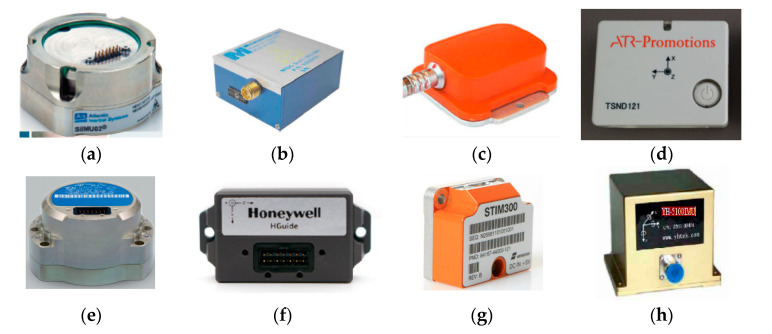
Some typical MIMUs used for pipeline surveying. (**a**) SiIMU02 IMU; (**b**) MIDG II IMU; (**c**) Xsens MTi IMU; (**d**) TSND121 IMU; (**e**) HG4930 IMU; (**f**) HGi300 IMU; (**g**) STIM300 IMU; (**h**) YH-5100 IMU.

For the intelligent PIG of small-diameter pipeline surveying, both the conventional mechanical gyroscope constructed PSINS and high-end FOG constructed SINS cannot be used directly because of their limitations in the measurement range and the dimensions of the small-diameter pipelines. Fortunately, with the rapid development of the MEMS sensor technology in recent years, the matured MEMS IMU could be installed inside the intelligent PIG and make possible pipeline surveying tasks, especially for the small-diameter pipeline [27,66,67,68,69,70,71,72,73,74]. Specifically, there are at least eight types of typical small-dimension MEMS IMUs used in the researching of the small-diameter intelligent PIG surveying, which are SiIMU02 IMU (Figure 6a), MIDG II IMU (Figure 6b), Xsens MTi IMU (Figure 6c), TSND121 IMU (Figure 6d), HG4930 IMU (Figure 6e), HGi300 IMU (Figure 6f), STIM300 IMU (Figure 6g) and the YH-5100 IMU (Figure 6h) [6,66,67,68,69,70,71,72,73,74].

However, their surveying precision cannot meet the precision requirements when only adopting the conventional integration algorithms usually based on the SINS, odometers, aboveground markers (AGMs) and the related intelligent PIG surveying technology. This is mainly because the small dimensions of the small-diameter pipeline limit the power supply and travel distance for each launch and receiving of the intelligent PIG. However, the MEMS IMU needs the distance of two adjacent AGMs less than 100 m to ensure the surveying precision, while the tunnels, aboveground forests, high buildings, roads, and rivers usually make the AGMs difficult to implement within 100 m [70,71]. Therefore, more characteristics of the routed pipeline should be used to enhance the surveying precision of the intelligent PIG.

## 4. Multi-Sensor Fused Small-Diameter Intelligent PIG Surveying Technologies

In this section, five types of small-diameter intelligent PIG surveying technologies based on the multi-sensor fused algorithm are introduced. Except for the traditional error estimation algorithms when using the high-precision MIMU and different measurement updates, these methods also utilize the pipeline characteristics, such as Pipeline Junction (PJ), Pipeline Segment Length (PSL) and the PBA to improve the surveying precision of the small-diameter intelligent PIG.

### 4.1. Strapdown Inertial Navigation System (SINS)/Odometer (Odo)/Above Ground Marker (AGM)-Based Intelligent PIG Surveying Technology

**Figure 7:**
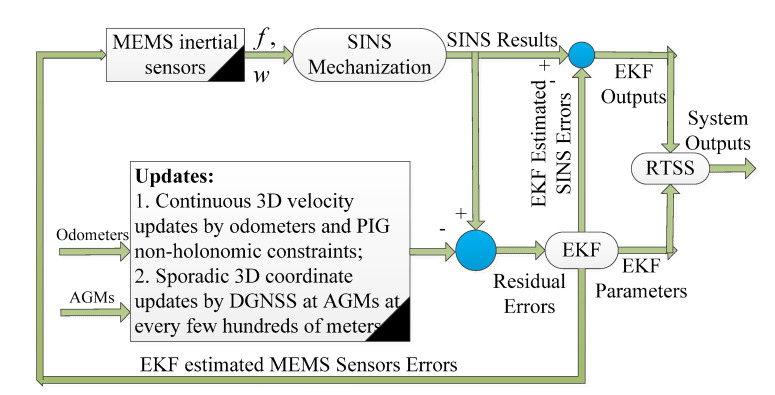
Schematic of Strapdown Inertial Navigation System (SINS)/Odometer (Odo)/Above Ground Marker (AGM)-based intelligent PIG surveying technology.

The first and often-used scheme of the intelligent PIG surveying technology is the SINS/Odo/AGM integration; its schematic diagram is shown in Figure 7 [66,67,68,69,70,71,72,73,74]. To be understood more clearly, the measurements of the micro-inertial sensors are utilized for SINS mechanization to calculate the pipeline central line coordinates and orientation continuously. At the same time, both the outputs of the odometers and the non-holonomic constraint characteristics of the intelligent PIG in the pipeline are combined for continuous 3D velocity updates. Furthermore, the AGMs with their coordinates provided by DGNSS at every few hundred meters are used for the sporadic 3D coordinate updates.

Generally, the Extended Kalman Filter (EKF) and the Rauch–Tung–Striebel Smoother (RTSS) are combined to implement the estimation of the SINS errors and the MIMU errors at the same time [75]. The EKF estimation algorithm process to calculate the current information in real-time from the first epoch to the current epoch, while the RTSS estimation algorithm usually calculates a point in an offline way by using all the measurement information from the first epoch to the last epoch [76,77]. Therefore, both the EKF and the RTSS are integrated to improve the surveying precision of the MIMU-based multi-sensor fused PIG system.

However, this technical scheme is viable when the inspected pipeline length is within few hundreds of meters and only by using the MIMU. The characteristics of the inspected pipeline are not fully used to improve the surveying precision of the intelligent PIG. The surveying precision of the intelligent PIG usually cannot reach the precision requirements especially when the diameter of the pipeline is less than 30 cm, which is mainly because the precision performances of the MIMU cannot meet the requirements when compared with the commonly used FOG-based SINS.

### 4.2. SINS/Odo/AGM/Pipeline Junction (PJ)-Based Intelligent PIG Surveying Technology

Pipeline Junctions (PJs), such as the valves, ring welds and the pipeline bending angles, are the key components that connect two adjacent Straight Pipeline Segments (SPS) or the Bent Pipeline Segments (BPS) [78,79]. PJs detection has been completely implemented by wavelet and fast orthogonal search algorithms when analyzing the MIMU data from the intelligent PIG [78,79]. Both the azimuth and pitch angles of the PIG surveying system within each independent SPS are invariant when they travel by using the cylindrical-shaped intelligent PIG, while the roll of the PIG is changed with its forward, irregular rotation within the overall inspected pipeline. Therefore, the azimuth and pitch angles calculated by the SINS mechanization at the beginning of each SPS could be adopted as the measurement updates of the filter and estimation algorithms for the corresponding SPS.

**Figure 8:**
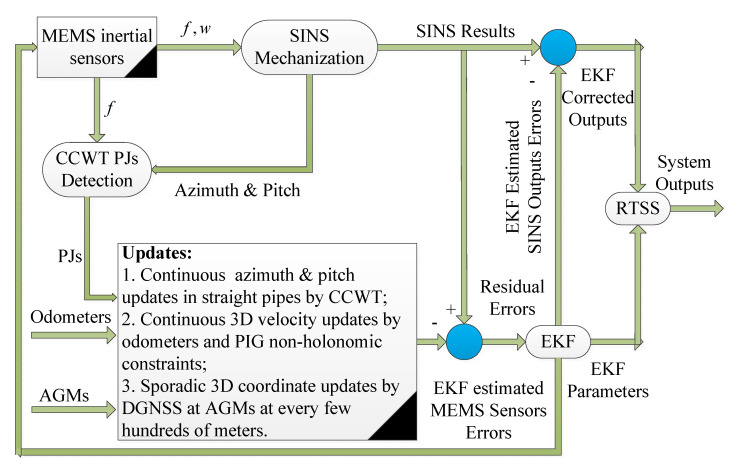
Schematic of SINS/Odo/AGM/Pipeline Junction (PJ)-based intelligent PIG surveying technology.

The second valuable scheme of the intelligent PIG surveying technology is the SINS/Odo/AGM/PJ integrated method. Its schematic diagram is shown in Figure 8 [36,80,81]. Firstly, the measurements of the micro-inertial sensors are used for SINS mechanization to obtain the pipeline central line coordinates and routing orientation continuously. After that, the measurements of odometers and the non-holonomic constraint properties of the intelligent PIG are integrated for the continuous 3D velocity updates. Moreover, the AGMs with coordinates provided by DGNSS at every few hundred meters are used for the sporadic 3D coordinate updates. Finally, the detection of the PJs is adopted for the continuous azimuth and pitch angles updates in each independent SPS. Hence, the SINS/Odo/AGM/PJ-based intelligent PIG surveying technology could improve the overall surveying precision by 50–70% in different research when compared with the previous SINS/Odo/AGM integration scheme [36,80,81].

### 4.3. SINS/Odo/PJ/Pipeline Segment Length (PSL)-Based Intelligent PIG Surveying Technology

When the aboveground environments of the inspected pipeline are covered by tunnels, forests, high buildings, roads, rivers and so on, the coordinates of the AGMs cannot be obtained accurately within a few hundreds of meters because of the DGNSS signal is totally blocked or interrupted by the surrounding environment. Fortunately, the Pipeline Segment Length (PSL) information is possible to be archived since the first routing of the overall pipeline. The PSL information can be obtained from the routing files and utilized for continuous 3D coordinate updates at the SPS part of the inspected pipeline. As such, the PSL can provide alternative information for the continuous 3D coordinate updates in the SPS part, especially when the AGMs coordinates are not available.

**Figure 9:**
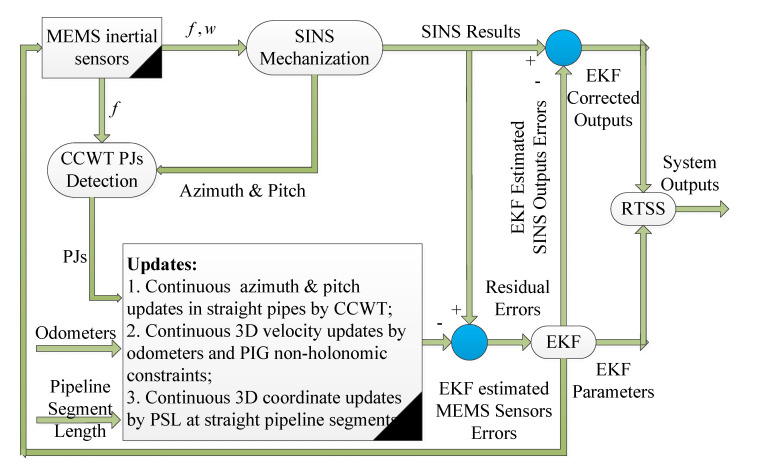
Schematic of SINS/Odo/PJ/Pipeline Segment Length (PSL)-based intelligent PIG surveying technology.

Based on the PSL information, the third scheme of the intelligent PIG surveying technology is the SINS/Odo/PJ/PSL integration structure. The corresponding schematic diagram is shown in Figure 9. More specifically, this technology integrates the measurements of the odometers and the non-holonomic constraint characteristics of intelligent PIG for continuous 3D velocity updates, the detection of PJs for continuous azimuth and pitch angles updates in the SPS part, and the information of PSL for continuous 3D coordinate updates in the SPS part. Therefore, the SINS/Odo/PJ/PSL integration scheme is an alternative scheme, especially when the coordinates of the AGMs cannot be obtained.

### 4.4. SINS/Odo/AGM/PJ/Pipeline Bending Angle (PBA)-Based Intelligent PIG Surveying Technology

PBA is another important property that could be adopted for the continuous azimuth and pitch angle updates at the PBA part and to improve the PBA detection precision and the overall surveying precision of the intelligent PIG. PBA information could be checked from the archived routing files of the inspected pipeline on one hand. On the other hand, the PBA could be calculated by combing the measurements of the symmetric installed odometers and the MIMU when the PIG travels through the PBA part [53,54].

**Figure 10:**
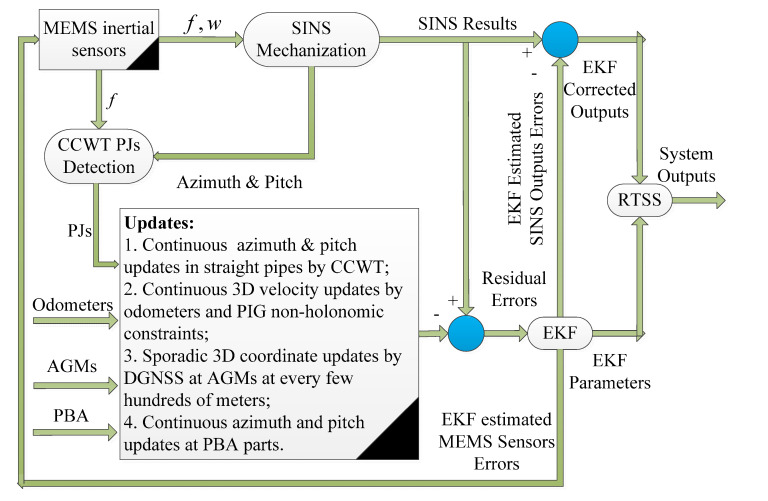
Schematic of SINS/Odo/AGM/PJ/Pipeline Bending Angle (PBA)-based intelligent PIG surveying technology.

The fourth scheme of the intelligent PIG surveying technology is the SINS/Odo/AGM/PJ/PBA integration, and its schematic diagram is shown in Figure 10. This technical scheme uses the measurements of odometers and the non-holonomic constraint properties of the intelligent PIG for continuous 3D velocity updates at first. Meanwhile, the detection of PJs for continuous azimuth and pitch updates occur in the SPS part; the information of AGMs provided by DGNSS for continuous 3D coordinate updates, and the calculation of PBA for continuous azimuth and pitch angles updates occur in the PBA part.

### 4.5. SINS/Odo/PJ/PSL/PBA-Based Intelligent PIG Surveying Technology

**Figure 11:**
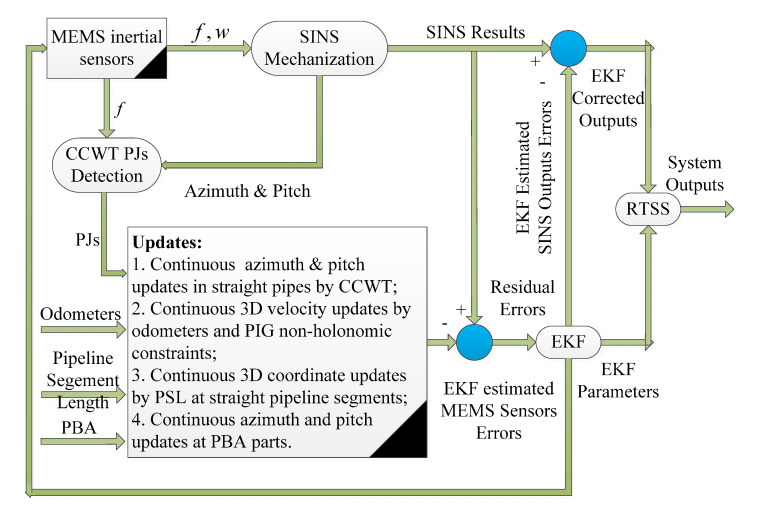
Schematic of SINS/Odo/PJ/PSL/PBA-based intelligent PIG surveying technology.

The fifth scheme of the intelligent PIG surveying technology is the SINS/Odo/PL/PSL/PBA integration; its schematic diagram is revealed in Figure 11. This technical scheme adopts the measurements of the odometers and the intelligent PIG non-holonomic constraints for continuous 3D velocity updates, including the detection of PJs for continuous azimuth and pitch angle updates in the SPS part, and the information of PSL for continuous 3D coordinate updates in the SPS part, and also the calculation of PBA for continuous azimuth and pitch angles updates in the PBA part.

## 5. Trends and Challenges for Small-Diameter Intelligent PIG Surveying Technologies

### 5.1. Advanced Inertial Sensors Technologies

At present, the micro-inertial sensors are also sensitive to ambient temperature variation and dynamic shocks and vibrations from complex environments when compared with other high-precision, large-dimension and expensive FOG-based inertial sensors [82,83]. However, even the modern and advanced inertial sensors, such as micro-inertial sensors, atomic gyroscope-based inertial sensors are developing. Their precision and reliability improve gradually because of the new error modelling technology of the micro-inertial sensors and the manufacturing technology in the atomic gyroscope [84,85,86]. Hence, the inertial sensors would be more immune to the complex application environments and their measurement precision should continue to improve.

Moreover, the redundant MIMU configuration is another way to improve the overall precision of the MIMU-based PIG surveying system, especially when the independent MIMU-based multi-sensor surveying system cannot satisfy the precision requirements [87,88]. Actually, both the precision and reliability of the redundant MIMU configurated PIG surveying system could improve, and the cost of the redundant inertial sensors and the dimensions would also increase to an acceptable range.

All in all, the precision of the small-diameter intelligent PIG surveying system would also improve with the accuracy enhancement of the advanced inertial sensors technology.

### 5.2. Modern Optimal Estimation Technology

Except for the traditional EKF signal estimation and processing technology for improving the surveying precision of the intelligent PIG, the nonlinear signal filter and estimation algorithms such as the Unscented Kalman Filter (UKF), Particle Filter (PF), Cubature Kalman Filter (CKF) and their adaptive estimation algorithms are widely used in the navigation of vehicles, shipborne and aerospace fields [89,90,91,92]. In addition, the Two-Filter Smoother (TFS) and the RTSS are also adopted for the offline process to improve the precision of the PIG surveying system [93,94,95]. Therefore, they are also potential optimal estimation technologies to solve the nonlinearity problems of the low-cost micro-inertial-based multi-sensor surveying system of the intelligent PIG.

In addition, with the rapid development and application of the Artificial Intelligent (AI) technology, the AI-related intelligent technology is also a potential research direction for the optimal estimation of the multi-sensor-fused intelligent PIG surveying system [96,97,98,99].

### 5.3. Challenges and Trends for Intelligent PIG Surveying Technology

The multi-sensor fused technology of intelligent PIG surveying is one of the key technologies to implement the PIM for small-diameter operating pipelines [100]. Specifically, the characteristics of the routed pipeline, such as the straight pipeline segment, pipeline segment length, pipeline bending angle and pipeline junction, should be fully utilized to correct and improve the surveying precision of the intelligent PIG, especially when adopting the small-dimension and the low-precision MIMU. Generally, the distributions of the small-diameter pipelines are mainly under the ground of urban areas, below the riverbed and refinery factory with a high density. These areas should be inspected regularly and carefully because of the high density of the population distribution and the complexity and intensity of the pipeline’s distribution. Therefore, the challenges for the intelligent PIG surveying technology are significant [101,102,103,104]:

(1) Some of the inspected pipelines cannot obtain precise coordinate information of the AGMs before the pigging operation, which is used to coordinate corrections of the intelligent PIG surveying system. This is mainly because the aboveground environment of these pipelines is occupied by the infrastructures such as buildings, roads, lakes, forests and so on. The GNSS signal is blocked or interrupted by these objects, so the precise coordinate of the AGMs cannot be provided.

(2) Some of the pipelines that can be surveyed by the intelligent PIG only have one entrance point and no exit point, so the intelligent PIG should be launched and received at the same entrance point. Meanwhile, the intelligent PIG surveying system only has the coordinate updates at the entrance point that would influence the overall intelligent PIG surveying precision.

(3) The surveying precision of the small-diameter intelligent PIG should be higher, especially when their surroundings are covered by pipelines, buildings, roads, rivers and so on. This is mainly based on the need for convenient repair and will not destroy any adjacent infrastructures as well.

(4) Modern digital and intelligent pipeline construction and routing technology requires PIG surveying technology to be fully digital and at least partially intelligent [105]. Therefore, the multi-sensor fused small-diameter pipeline surveying technologies will play important roles in the near future.

(5) The integration of modern digital and intelligent pipeline technology, small-diameter pipeline inspection and surveying technologies, the Geographic Information System (GIS) [106,107,108], satellite remote sensing technology, and aboveground Unmanned Autonomous Vehicle (UAVs) detection technology are the major trends in the development of intelligent pipelines [109,110,111].

## 6. Conclusions and Future Work

Small-diameter pipelines account for approximately 1/3 of the total routed pipelines, which are used for different types of energies transportation. This paper introduced a comprehensive review of MIMU-based intelligent PIG multi-sensor fusion technologies for small-diameter pipeline surveying. The existing and most popular four kinds of intelligent PIGs and their corresponding pipeline defect inspection technologies for small-diameter pipeline surveying were reviewed. In addition, two types of intelligent PIG surveying technologies, non-inertial-based and inertial-based, were introduced. Then, five different schematic diagrams of the intelligent PIG surveying methods were demonstrated for different applications. Finally, the trends and challenges of small-diameter pipeline surveying technologies are also analyzed and revealed in this paper. Therefore, to implement the PIM and the safety operation of the routed small-diameter pipelines are of great importance to reduce the overall cost and negative social influence caused by the pipeline leakages or even explosions.

As for the future work, we will further investigate low-cost multi-sensor fused small-diameter pipeline surveying technologies that focus on optimal estimation algorithms and new types of pipeline structures, as well as the redundant MIMU configuration-based multi-sensor fused intelligent PIG surveying system.

## Figures and Tables

**Table 1:** The comparison between various pipeline surveying technologies.

	Surveying Technologies	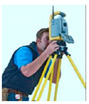		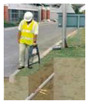		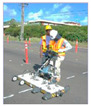		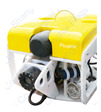		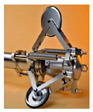
Pipeline Types		Conventional Optical Station Surveying		Walkover Beacon-Based Surveying		Ground Penetrating Radar Surveying		ROV/AUV-Based Multi-Sensor Surveying		Multi-Sensor Fused Intelligent PIG Surveying
Land Pipelines	Existing Pipeline			OSDP		OSDP				Viable
			
	Newly Trenched Pipeline	Viable		High costs		High costs				Viable
	
	Trenchless Pipeline			OSDP		OSDP				Viable
			
Marine Pipelines	Existing Pipeline							OSDP with HC		Viable
					
	Newly Trenched Pipeline							OSDP with HC		Viable
					
	Trenchless Pipeline							OSDP with HC		Viable
					

Where, ‘\’ denotes this technology is “not viable” for this type of pipeline; ‘OSDP’ denotes “Only for shallow depth pipeline”; ‘HC’ denotes “high costs”.

**Table 2:** Summary of interior pipeline leakage detection technologies.

Technologies		Principle of Operation	Strengths	Weaknesses
**Sonar**	Ultrasonic	An ultrasonic signal is transmitted, reflected off the walls, and received again by the sonar head. The flight time is used to compute the distance, and an internal profile is determined.	Provides information on any deformation and the existence of cracks.	Its inability to inspect both the flooded and the dry parts of the pipeline.
**Electromagnetic sensors**	MFL	Measuring the disturbances of the magnetic flux with a Hall-effect device. Disturbances on the flux are caused by the defects in the pipe material. Also, the wall thickness can be determined by analyzing the induced magnetic flux.	Cracks, leaks, corrosion pits and wall thickness can be determined	Energy consumption is high
	Remote Field Eddy Current (RFEC)	A solenoid exciter coil is used to create an electromagnetic field generating eddy currents and magnetic flux lines within the pipe. Sensors positioned in the remote field region can detect minor variations in the field.	Detection of the remaining wall thickness and the location of corrosion pitting and axial cracks.	Energy consumption is high
	Remote Field Transformer Coupling (RFTC)	It detects any broken wires in the Pre-stressed Concrete Cylinder Pipe (PCCP). It detects of breaks in the pre-stressing wires on PCCP and holes or perforations within the steel cylinder core used in the PCCP construction.	Detect and locate any broken wires, manholes and joints.	Energy consumption is high
	Broadband Electromagnetic (BEM)	Based on transmitting a signal that covers a broad-frequency spectrum.	Located and reported cracks, fractures, and pipe wall thickness.	Energy consumption is high
	Microwave Backscattering Sensor (MBS)	Generated microwaves penetrate nonmetallic pipe and backscattered by material changes. By evaluating the phase shift and the amplitude between emitted and received microwaves.	Detect the material inhomogeneity.	Cannot penetrate water or reinforced concrete pipes.
	Ground Penetrating Radar (GPR)	Based on emitting pulsed microwaves with varying frequencies, allowing a varying penetration depth and measurement resolution. 3D GPR images can be produced by raw field data and post-processing software.	Some solutions exist for pipe GPR providing material inhomogeneity in the pipe bedding.	The inspection depth of the pipeline is limited.
**Optical sensors**	Laser profiler	Provides very good accuracy in the estimation of the pipe geometry	Detection ovality, pipe deformation and geometry with good accuracy.	Energy consumption is high.
	Closed Circuit Television (CCTV)	Several different cameras available for in-pipe inspection robots such as the fish-eye concept.	Detection of ovality or pipe deformation is possible.	Weak light environment is impossible.
